# Complete genome analysis and antimicrobial mechanism of *Bacillus velezensis* GX0002980 reveals its biocontrol potential against mango anthracnose disease

**DOI:** 10.1128/spectrum.02685-24

**Published:** 2025-04-16

**Authors:** Jing Wang, Xiaoying Xie, Bo Li, Lifang Yang, Fuqiang Song, Yan Zhou, Mingguo Jiang

**Affiliations:** 1 Guangxi Key Laboratory for Polysaccharide Materials and Modifications, School of Marine Sciences and Biotechnology, Guangxi Minzu University 47874 , Nanning, Guangxi, China; 2 Guangxi Key Laboratory for Polysaccharide Materials and Modifications, School of Chemistry and Chemical Engineering, Guangxi Minzu University 47874 , Nanning, Guangxi, China; 3 Engineering Research Center of Agricultural Microbiology Technology, College of Heilongjiang Province & School of Life Sciences, Heilongjiang University 12432, Harbin, Heilongjiang, China; The Hebrew University of Jerusalem, Rehovot, Israel

**Keywords:** *Bacillus velezensis*, *Colletotrichum gloesporioides*, whole genome sequencing, secondary metabolite gene cluster, liquid chromatography-mass spectrometry

## Abstract

Post-harvest anthracnose significantly affects the yield and quality of mangoes. In this study, an antagonist bacterium, GX0002980, with strong inhibitory effect against *Colletotrichum gloesporioides*, the pathogen of mango anthracnose, was isolated from the rhizosphere soil of plants. Based on morphological characteristics, physiological and biochemical tests, and 16S rRNA gene and *gyrB* gene sequencing analysis, the strain was identified as *Bacillus velezensis*. Strain GX0002980 exhibits broad-spectrum antibacterial capabilities, and its volatile substances and sterile fermentation filtrate also demonstrate antagonistic effects against *C. gloesporioides*. This strain can produce antimicrobial substances that inhibit pathogen growth, such as amylase, cellulase, protease, pectinase, and siderophores, and has plant-growth-promoting characteristics, such as nitrogen fixation, NH_3_ production, and phosphate solubilization. Whole-genome sequencing results show that the genome size of GX0002980 is 3,907,381 bp with a guanine and cytosine content of 47.44%. The antiSMASH analysis predicts 14 antimicrobial biosynthesis gene clusters within the GX0002980 genome, including surfactin, fengycin, bacilysin, macrolactin H, bacillaene, difficidin, and bacillibactin. Liquid chromatography-mass spectrometry analysis revealed that the antimicrobial active substances secreted by GX0002980 include surfactin, bacilysin, butirosin A, and more. Strain GX0002980 has an *in vitro* control efficiency of 52% against mango anthracnose, and it can effectively suppress the occurrence of post-harvest diseases in mangoes, extending their storage time. In conclusion, *B. velezensis* GX0002980 possesses multiple biocontrol mechanisms and has potential for application in the biological control of mango anthracnose.

**IMPORTANCE:**

*Bacillus velezensis* GX0002980 showed biocontrol potential against *Colletotrichum gloesporioides*, a causative agent of mango anthracnose. *B. velezensis* GX0002980 produces a variety of secondary metabolites with antibacterial properties. Whole-genome sequencing revealed potential active metabolite synthesis gene clusters of *B. velezensis* GX0002980. *B. velezensis* GX0002980 has a significant effect on the control of post-harvest disease in mango fruits.

## INTRODUCTION

Mango (*Mangifera indica* L.) is one of the most economically valuable tropical fruits in the world and is known as the “king of tropical fruits” (1). Mango cultivation is widespread, with production in over 70 countries, 90% of which is concentrated in Asian countries such as India, China, Pakistan, and Bangladesh (2). Mango anthracnose is one of the most severe global diseases in mangoes, occurring both during cultivation and post-harvest storage, characterized by dark brown spots on the fruit’s skin and soft rot in the flesh. Anthracnose accounts for over 60% of the total mango disease burden during storage, significantly reducing the quality of the fruit and causing substantial economic losses, severely limiting the sustainable and healthy development of the mango industry. The disease is primarily caused by the infection of *Colletotrichum gloesporioides* (3). Besides mangoes, *C. gloesporioides* can also infect a variety of fruits, such as bananas, longan, apples, papayas, and citrus (4, 5). Recently, *C. gloesporioides* has become a global problem, causing significant economic losses in agricultural production (3).

Currently, the control of anthracnose is mainly based on chemical treatments, such as fungicides like benomyl, prochloraz, thiabendazole, and mancozeb, which are applied by soaking or spraying the fruit before harvest. However, there are many issues with these methods, including high cost, environmental pollution, pesticide residues, and pathogen resistance (6, 7). Biocontrol bacteria, due to their green safety, broad application prospects, and low cost, have become a research hotspot for the control of anthracnose. Microbial control, as a new means to replace chemical fungicides, has been extensively studied. Research has shown that microbial control is effective in the control of post-harvest anthracnose in fruits (8). To date, it has been reported that *Papiliotrema aspenensis* DMKU-SP67 has a good control effect on banana anthracnose, reducing the incidence rate by 94.1%, which is comparable to the control effect of phenol (93.9%) (9). The metabolic products of *Bacillus atrophaeus* B5 have an inhibitory effect on the spore germination of the pathogen *C. gloesporioides* in soursop and avocado post-harvest anthracnose (10). Li et al. (11) isolated a strain, *Bacillus safensis* sp. QN1NO-4, from Noni (Morinda Citrifolia) that exhibits strong antagonistic activity against strawberry anthracnose and successfully inhibits the infection of *C. gloesporioides* on post-harvest strawberry fruits. As a biocontrol bacterium, *Bacillus* sp. can control a variety of microbial diseases and has great potential in improving agricultural productivity (12). *Bacillus* sp. prevents plant diseases by producing biologically active secondary metabolites. The antibacterial substances secreted by *Bacillus* sp. mainly include lipopeptide compounds, polyketones, antibacterial proteins, and volatile compounds. Lipopeptide compounds are produced through a typical non-ribosomal synthesis pathway (NRPS) and are also known as lipopeptide antibiotics (13). Lipopeptide compounds such as surfactin, iturin, and fengycin are the main antibacterial active substances of *Bacillus* (14). These substances primarily act by altering the structure of the cell membranes of plant pathogens, reducing their functionality and permeability to inhibit the growth of pathogens. Polyketone compounds mainly include macrolactin, bacillaene, and difficidin. Antibacterial proteins are synthesized via the ribosomal pathway and have a twofold mechanism of action: one, antibacterial proteins possess cell wall-degrading enzymes such as chitinase, protease, and cellulase, which act on the fungal mycelium or spore cell walls, causing the mycelium to become distorted or inhibiting spore germination; and two, they can destroy the integrity of the cell membrane, causing the mycelial contents to leak out, leading to mycelial death. Additionally, volatile substances produced during the metabolic process of *Bacillus* have the ability to promote plant growth and inhibit plant pathogenic fungi (15). In this study, the pathogenic bacterium *C. gloesporioides* of mango anthracnose was used as the target fungus. A biocontrol strain, GX0002980, was isolated from the rhizosphere soil, and its whole genome was sequenced to explore its potential active metabolite synthesis gene clusters. By preparing antimicrobial active substances through crude extraction with ethyl acetate and combining liquid chromatography-mass spectrometry (LC-MS) for identification of these substances, the study further analyzes the biocontrol potential of strain GX0002980 and predicts and elucidates the antimicrobial mechanism, providing a theoretical basis for the development and application of strain GX0002980 and the biological control of post-harvest diseases in mangoes.

## MATERIALS AND METHODS

### Source of the samples

Soil samples were collected from the Damingshan Nature Reserve in the Guangxi Zhuang Autonomous Region (23°37′ N, 108°49′ E, elevation 546.8 m). The test pathogenic fungi were obtained from the Guangxi Polysaccharide Materials and Modification Key Laboratory, including *Colletotrichum gloeosporioides*, *Colletotrichum fructicola*, *Colletotrichum asianum*, *Colletotrichum siamense*, *Fusarium chlamydosporum*, *Botryosphaeria dothidea*, *Fusarium oxysporum*, *Bipolaris sorokiniana*, *Fusarium pseudograminearum*, *Plectosphaerella cucumerina*, *Alternaria alternata*, *Rhizoctonia solani*, and *Cryphonectria parasitica*.

### Isolation and screening of rhizosphere bacteria in rhizosphere soil

Ten grams of soil samples was added to 90 mL of sterile water and shaken at 30°C at 200 rpm for 3 h. Afterward, the soil suspension was gradient-diluted to 10^−3^, 10^−4^, and 10^−5^ dilutions, and 100 µL of each dilution was spread evenly on lysogeny broth (LB) solid medium and then incubated inverted at 30°C. After 24 h, colonies of different sizes and morphologies were picked and streaked onto new LB solid plates for purification. The strains were inoculated in 20 mL of LB liquid medium and shaken at 30°C at 200 rpm for 24 h to obtain the fermentation broth. The fermentation broth was centrifuged at 8,000 rpm for 5 min; the bacteria were dispersed with sterile water and then centrifuged at 8,000 rpm for 5 min and repeated twice. A standard bacterial suspension with OD_600_ ≈ 1.0 was prepared with sterile water and set aside.

Referring to the method of Li et al. (16), we determined the inhibitory activity of the antagonistic bacterial strain against the target fungus, *C. gloeosporioides,* by plate confrontation. A 6 mm diameter pathogen fungal cake was inoculated at the center of a potato dextrose agar (PDA) plate, and 5 µL of the bacterial suspension was inoculated 2.5 cm from the center of the plate. A PDA media with only *C. gloeosporioides* served as the control. The experiment was repeated three times, and the sample was incubated at 28°C for 7 days to observe the inhibitory effect and calculate the inhibition rate (17).


$$Inhibition\;Rate(\%)=\frac{Colony\;Diameter\;of\;Control\;Group\;(mm)-Colony\;Treatment\;of\;Control\;Group\;(mm)}{Colony\;Diameter\;of\;Control\;Group\;(mm)}\times 100$$

Morphological identification and housekeeping gene amplification

Following the methodology of Balows (18), the antagonistic bacterial strain was observed morphologically and identified through various physiological and biochemical tests, including Gram staining, oxidase testing, starch hydrolysis, gelatin liquefaction, methyl red test, and litmus milk tests.

Total DNA was extracted from the activated antagonistic strain and used as a template for PCR amplification of the 16S rRNA and *gyrB* genes using universal bacterial primers 27F/1492R (5′-AGAGTTTGATCCTGGCTCAG-3′ and 5′-TACGGCTACCTTGTTACGAGTT-3′) (19) and UP-1/UP-2r (5′-GAAGTCATCATGACCGTTCTGCAYGCNGGNGGNAARTTYGA-3′ and 5′-AGCAGGATACGGATGTGCGAGCCRTCNACRTCNGCRTCNGTCAT-3′) (20).

The PCR amplification system (50 µL) consisted of 1 µL template, 22 µL ddH_2_O, 25 µL 2× Taq PCR Mix, 1 µL forward primer, and 1 µL reverse primer. The PCR program included an initial denaturation at 95°C for 5 min, followed by 32 cycles of denaturation at 95°C for 1 min, annealing at 55°C for 1 min, and extension at 72°C for 2 min, with a final extension at 72°C for 10 min, and storage at 4°C. The amplified products were sequenced by Sangon Biotech Co., Ltd. (Shanghai, China), and sequences were compared with those in the National Center for Biotechnology Information (NCBI) database for homology. Phylogenetic trees were constructed using Phylosuite v.1.2.3 software, and the phylogenetic tree was visualized and adjusted using Fig Tree v.1.4.4 software. The 16S rRNA (PV239417) and *gyrb* gene (PV254805) sequences have been uploaded to the NCBI.

### *In vitro* study

The inhibitory effect of volatile compounds produced by *Bacillus velezensis* GX0002980 on *C. gloeosporioides* was determined using a double-plate method. *B. velezensis* GX0002980 was inoculated in 50 mL of LB liquid medium and shaken at 30°C at 200 rpm for 24 h. One hundred microliters of the fermentation broth was spread evenly on an LB solid plate, and a 6 mm *C*. *gloeosporioides* cake was placed at the center of a PDA plate. The two plates were sealed face to face and incubated at 28°C for 7 days. The control group consisted of double plates without the inoculation of *B. velezensis* GX0002980 culture.

The inhibitory effect of the sterile fermentation filtrate of *B. velezensis* GX0002980 on *C. gloeosporioides* was determined by the growth rate method. *B. velezensis* GX0002980 was inoculated in 50 mL of LB liquid medium and shaken at 30°C at 200 rpm for 24 h. The fermentation broth was centrifuged at 10,000 rpm for 20 min, and the supernatant was filtered through a 0.22 µm sterile filter membrane twice. The filtrate was added to the PDA plate at 50°C at proportions of 1%, 2%, 5%, 10%, and 20%, mixed thoroughly, and poured into plates. A PDA medium without a sterile fermentation filtrate (0%) served as the control, with each treatment replicated three times. After the medium solidified, a 6 mm *C*. *gloeosporioides* fungal cake was inoculated at the center and incubated at 28°C for 7 days before the colony diameter was measured to calculate the inhibition rate.

### Mycelial morphology and hyphal ultrastructure

The 1 cm^2^ mycelium of *C. gloeosporioides* from the edge of the inhibition zone was cut and compared with the mycelium of *C. gloeosporioides* grown on normal medium (21). The morphological changes in the mycelium were observed through a scanning electron microscope.

### Evaluation of the antibacterial spectrum of antagonistic bacteria

Referring to the aforementioned plate confrontation method, the inhibition effect of the antagonistic bacteria on 12 common plant pathogenic fungi was determined, including *Fusarium graminearum*, *Botryosphaeria dothidea*, *Bipolaris sorokiniana*, *Fusarium pseudograminearum*, *Plectosphaerella cucumerina*, *Alternaria alternata*, *Rhizoctonia solani*, *Cryphonectria parasitica*, *Colletotrichum fructicola*, *Colletotrichum asianum*, *Colletotrichum siamense*, and *Fusarium chlamydosporum*. The inhibition rate was calculated.

### Study on the hydrolase activity of strain GX0002980

Five microliters of the bacterial suspension of the antagonistic bacteria (OD_600_ ≈ 1.0) was dropped onto the respective plates for amylase, protease, cellulase, and pectinase, and then incubated at 30℃ for 2–3 days. For the amylase activity test, the medium was stained with iodine solution; for the cellulase and pectinase activity tests, the medium was stained with 1 mol/L Congo red for 30 min, followed by decolorization with 1 mol/L NaCl solution for another 30 min. The presence of a transparent zone around the bacterial colony indicated enzyme activity.

### Research on plant growth-promoting characteristics

#### Nitrogen fixation

The antagonistic bacterial strain was streaked onto Ashby’s nitrogen fixation medium and incubated at 30°C for 3 days. Observation of normal growth on the plate indicates the ability of the strain to fix nitrogen.

#### Ammonia production

Fifty microliters of the antagonistic bacterial suspension (OD_600_ ≈ 1.0) was inoculated into a test tube containing 10 mL of peptone water medium. After shaking the culture at 30°C for 48 h, 0.5 mL of Nessler’s reagent was added. A change in the color of the solution from yellow to reddish brown with precipitate formation indicated the strain’s ability to produce NH_3_ (22).

#### Phosphate solubilization

Five microliters of the antagonistic bacterial suspension (OD_600_ ≈ 1.0) was inoculated onto the center of bacterial organophosphorus media and inorganic phosphate media. After incubation at 30°C for 5 days, the presence of a clear zone around the bacterial growth indicated the ability to solubilize organic or inorganic phosphate.

#### Siderophore production

The activated antagonistic strain was inoculated into the center of a medium designed for siderophore detection and incubated at 30°C for 5–7 days. The formation of an orange-yellow halo around the bacterial growth indicated the ability to produce siderophores; the size of the halo can be used to determine the capacity of the strain to produce siderophores.

#### IAA production

Following the method of AL-Habib (23), 10 µL of bacterial suspension (OD_600_ ≈ 1.0) was inoculated into the LB liquid medium containing 100 mg/L L-tryptophan. After shaking at 200 rpm at 30°C for 3 days, the culture was centrifuged at 8,000 rpm for 10 min. Then, 0.5 mL of the supernatant was mixed with an equal volume of Salkowski’s reagent. A 5 µg/mL IAA standard and a blank medium were used as positive and negative controls, respectively. After coloring in the dark for 30 min at room temperature, a red color change indicated IAA production. The OD value at 530 nm was measured, and the IAA content secreted by the strain was calculated based on the standard curve.

### Whole-genome sequencing, assembly, and annotation

After the extraction of genomic DNA (24) and its quality assessment, the concentration of the DNA sample was detected using a Qubit fluorescence quantitative analyzer; 1% agarose gel electrophoresis detected the integrity of the DNA samples. The Qubit dsDNA HS Assay Kit 500 assays were used to detect the amount of purified DNA sample. The genome was fully sequenced once purity and concentration met the sequencing requirements. Genes were predicted using Glimmer (version 3.02) software for assembly. rRNA was predicted using RNAmmer (version 1.2) software; tRNA regions and secondary structures were predicted using tRNAscan-SE (version 1.3.1) software; sRNA was obtained by comparing with the Rfam database using Infernal (version 9.1) software. Gene annotation was completed using Diamond, with comparison results in basic local alignment search tool tabular format. The sequencing results were annotated for gene function against 11 databases, including Gene Ontology (GO), Kyoto Encyclopedia of Genes and Genomes (KEGG), and Cluster of Orthologous Groups of proteins.

The secondary metabolite biosynthesis gene clusters in the genome of the antagonistic bacterium GX0002980 were predicted and analyzed using antiSMASH software (version 5.1.2), obtaining the predicted gene cluster information for the respective strains.

The average nucleotide identity (ANI) and the digital DNA-DNA hybridization (dDDH) were analyzed using Jspecies (http://jspecies.ribohost.com/jspeciesws/) and the Genome-to-Genome Distance Calculator (https://ggdc.dsmz.de/ggdc.php) to further determine the taxonomic status of GX0002980. Additionally, the secondary metabolite gene cluster of *B. velezensis* was compared.

### Extraction of antimicrobial active substances from *Bacillus velezensis* GX0002980 and identification of antimicrobial substances by LC-MS

Antimicrobial substances from the fermentation filtrate of GX0002980 were extracted using organic solvents. The fermentation filtrate of *B. velezensis* GX0002980 was mixed with an equal volume of ethyl acetate in a separatory funnel, shaken well, and left to stand for phase separation. After repeated shaking and standing three times, the upper organic phase was collected. The lower aqueous phase was extracted with ethyl acetate three times. The organic phase obtained from the extraction was concentrated in a rotary evaporator and dissolved in 4 mL of chromatographic grade methanol, then filtered through a 0.22 µm sterile filter membrane to obtain the crude extract. The antimicrobial activity of the crude extract of *B. velezensis* GX0002980 was tested against *C. gloeosporioides* using the Oxford cup method (25). The antimicrobial active substances of *B. velezensis* GX0002980 were analyzed and identified using liquid chromatography-mass spectrometry.

Chromatographic conditions were as follows: the chromatographic column was an Acquity UPLC HSS T3 (2.1 mm × 100 mm, 1.8 µm); mobile phase A was a 0.1% formic acid aqueous solution, and mobile phase B was a 0.1% formic acid acetonitrile; the flow rate was 0.40 mL/min; the injection volume was 1 µL; and the column temperature was 40°C. The analytical platform used was an ultrahigh-performance liquid chromatography system (Acquity I-Class PLUS; Waters Corporation, Milford, USA) coupled with a high-resolution mass spectrometer (Xevo G2-XS QTOF, Waters). Mass spectrometry signals were collected in both positive and negative ion scanning modes. The raw data were processed and identified using metabolomic processing software Progenesis QI (version 2.1) (Waters Corporation).

### Control and preservation effects of mango anthracnose disease during post-harvest storage

The antagonistic bacterial strain’s effect on the control of mango anthracnose was measured using a detached mango inoculation test. Following the method of Mangoba and Alvindia (26), Guiqi mangoes (80% maturity) were wiped with 75% ethanol solution, then repeatedly rinsed with sterile water, air-dried, and sprayed with a suspension of GX0002980 bacterial cells on the mango surface until droplets flowed down. After air-drying, the spraying operation was repeated three times. Sterile water was used as a control. The mangoes were soaked in *C. gloeosporioides* spore suspension with a concentration of 10^7^ conidia/mL for 5 min and then air-dried. They were then maintained at 25°C with humidity and observed for disease development after 7 days.

The effects of fermentation broth, bacterial suspension, sterile fermentation filtrate, and volatile gases of strain GX0002980 on the post-harvest storage of Guiqi mangoes were measured using the detached mango inoculation test as described above. The condition of the mangoes was observed after 12 days.

## RESULTS

### *In vitro* antimicrobial activity of strain GX0002980

**Fig 1:**
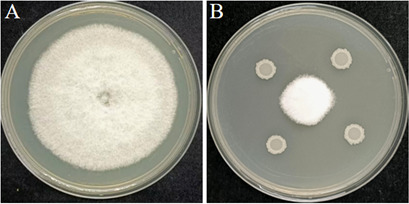
Antifungal effect of the antagonist bacterium GX0002980 against *C. gloeosporioides*: (**A**) control and (**B**) treatment.

A total of 46 morphologically distinct bacteria were isolated from the rhizosphere soil of plants. Using *C. gloeosporioides* as the target pathogen, we found that the plate confrontation method revealed strain GX0002980 to have the strongest inhibitory effect on *C. gloeosporioides*, with an inhibition rate as high as 70.98%. Therefore, the biocontrol strain GX0002980 was selected for subsequent research (Fig. 1).

### Morphological identification and housekeeping gene analysis

**Fig 2:**
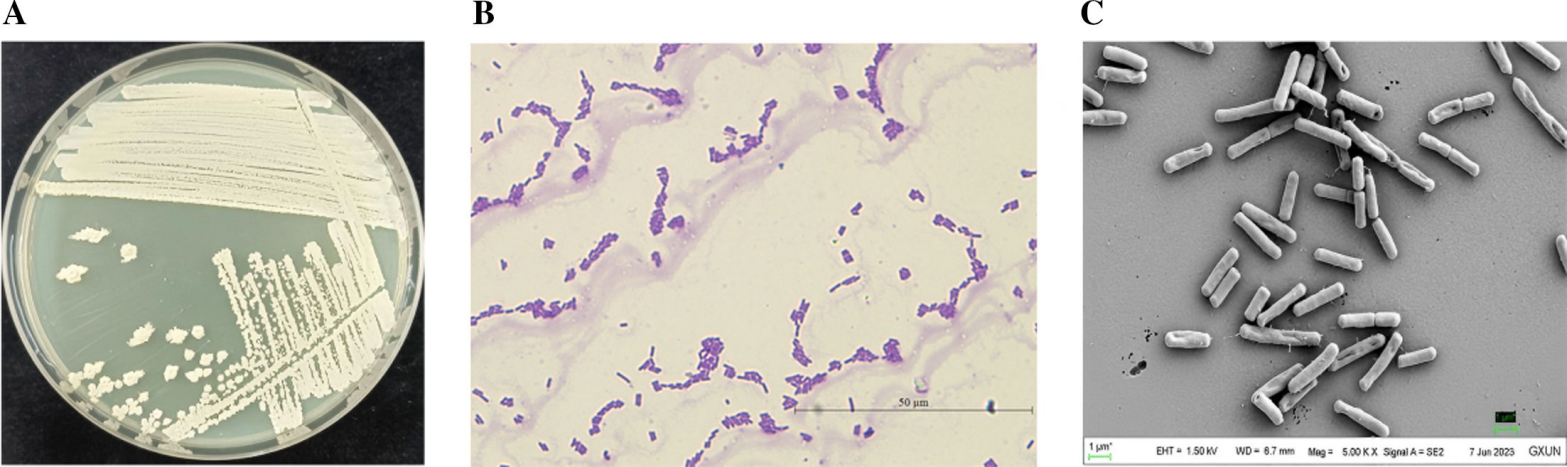
Morphological identification of the antagonistic bacterium GX0002980: (**A**) colony morphology of GX0002980 on LB medium, (**B**) gram staining, and (**C**) observation of morphology under a scanning electron microscope.

The antagonistic bacterium GX0002980 is a rod-shaped, gram-positive bacterium that forms white, dry, rough, and opaque colonies with wrinkles and bulges on an LB agar plate (Fig. 2). It is an aerobic bacterium. Indole, oxidase, hydrogen sulfide, methyl red, and litmus milk test results were negative; while starch hydrolysis, gelatin hydrolysis, urease, and citrate utilization test results were positive (Table 1).

**TABLE 1:** The results of physiological and biochemical experiments

Item	Result^*a*^
Anaerobic test	−
Starch hydrolysis	＋
Gelatin hydrolysis	＋
Indole	−
Oxidase	−
Hydrogen sulfide test	−
Methyl red test	−
Citrate salt	＋
Urease	＋
Litmus milk test	−

^*a*^ −, negative; +, positive.

**Fig 3:**
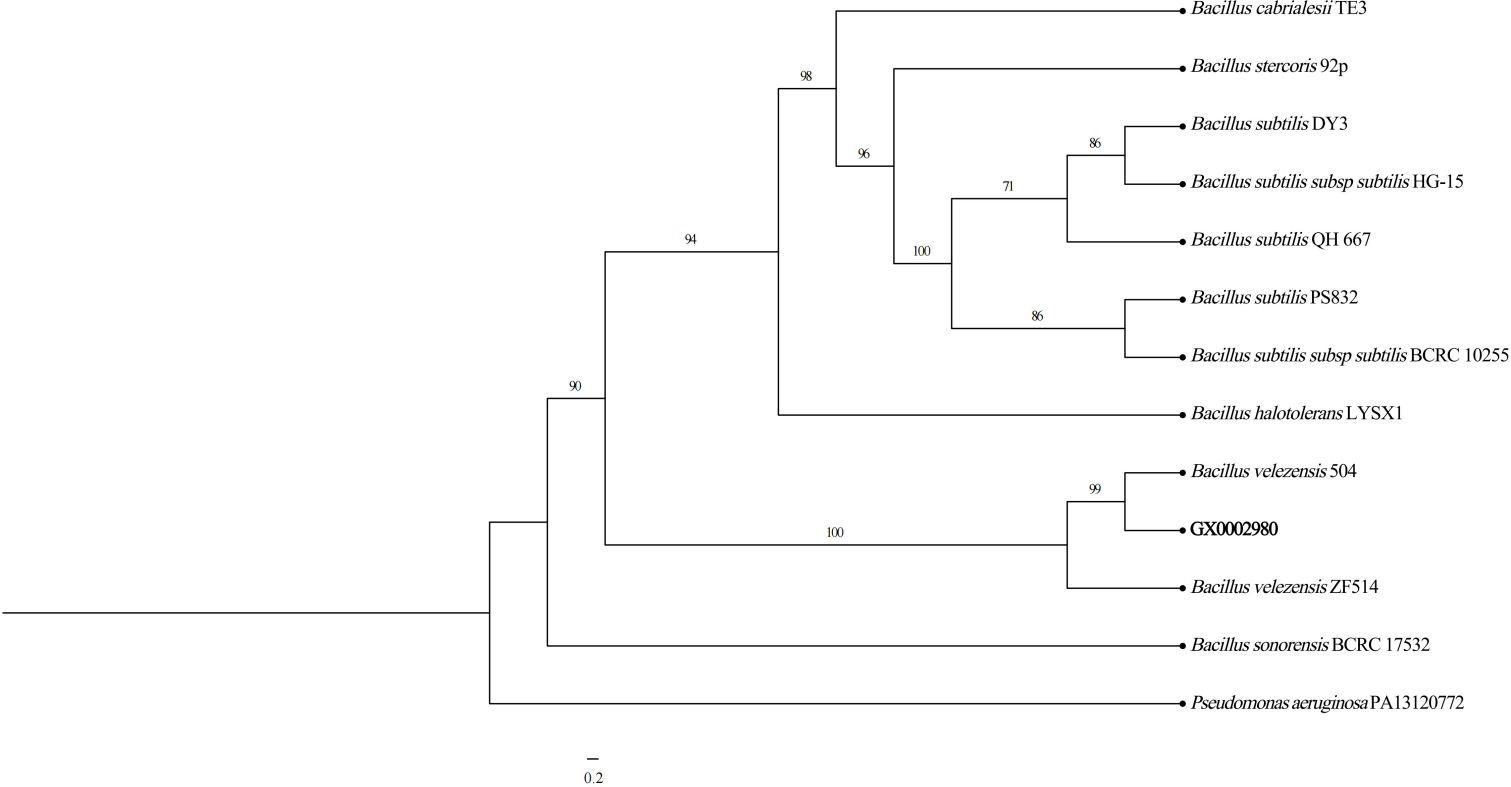
Multigene joint phylogenetic analysis of the antagonistic bacterium GX0002980 by Phylosuite v.1.2.3.

The 16S rRNA and *gyrB* gene sequences of strain GX0002980 were compared for homology with existing data in the NCBI database, and a phylogenetic tree was constructed (Fig. 3). The results showed that GX0002980 had a high similarity with *Bacillus* strains in the 16S rRNA gene results, with a similarity of over 99% with *Bacillus amyloliquefaciens* and *Bacillus velezensis*. The *gyrB* gene results showed that GX0002980 had a similarity of 100% with *Bacillus velezensis* 504. Using two gene sequences for multigene joint construction, we found that GX0002980 clustered with *Bacillus velezensis* 504, and the phylogenetic tree also clustered into one branch. Integrating the morphological characteristics, physiological and biochemical properties, and molecular biological identification results, the strain was identified as *Bacillus velezensis*.

### The inhibitory effects of the antagonist bacterium GX0002980 on the growth of *C. gloeosporioides*

**Fig 4:**
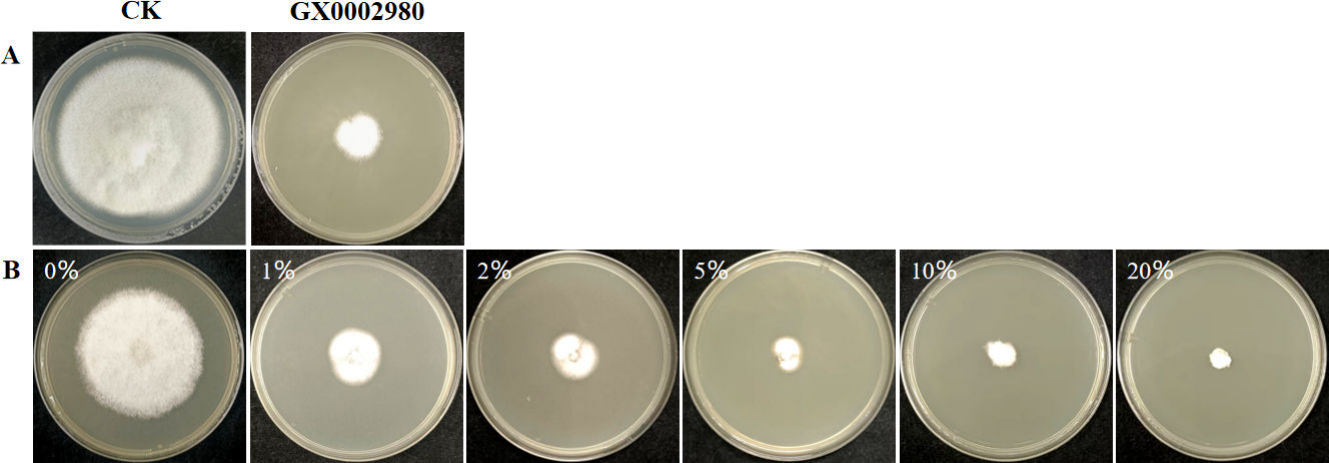
The inhibitory effects of the antagonist bacterium GX0002980 on the growth of *C. gloeosporioides*. (**A**) The inhibitory effect of volatile organic compounds on *C. gloeosporioides*. (**B**) The inhibitory effect of sterile fermentation filtrate on *C. gloeosporioides*.

The volatile compounds produced by the biocontrol strain GX0002980 also had an inhibitory effect on the mycelial growth of *C. gloeosporioides*, with an inhibition rate of 73.91%(Fig. 4A).

The sterile fermentation filtrate of biocontrol strain GX0002980 inhibited the mycelial growth of *C. gloeosporioides* at concentrations of 1%, 2%, 5%, 10%, and 20%, and the inhibition rate increased with the concentration of the GX0002980 sterile fermentation filtrate. At a GX0002980 fermentation filtrate concentration of 20%, the inhibition rate was close to 100% (Fig. 4B).

### Microscopic observation of the effect of GX0002980 on the mycelial growth of *C. gloeosporioides*

The results of scanning electron microscopy showed that strain GX0002980 had a significant inhibitory effect on mycelial growth of *C. gloeosporioides*.

**Fig 5:**
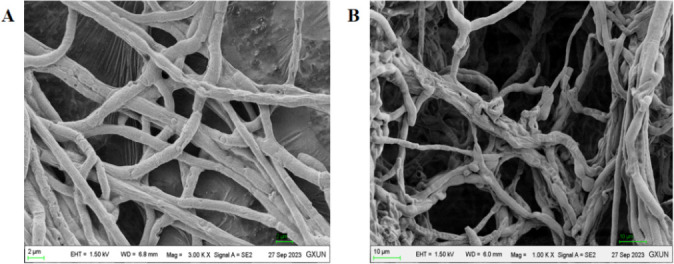
Scanning electron microscope observation of the effect of strain GX0002980 on the morphology of *C. gloeosporioides* mycelium: (**A**) control and (**B**) treatment.

In the control group, the amount of pathogen mycelium was abundant, uniform in thickness, and smooth in surface, while in the treatment group, the pathogen mycelium was severely deformed, showing signs of reduced quantity, shrinkage, distortion, and entanglement (Fig. 5).

### Antimicrobial spectrum determination of strain GX0002980

**Fig 6:**
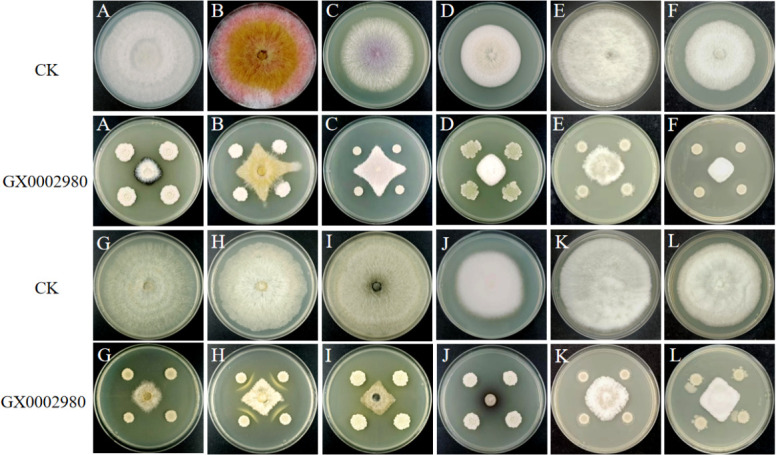
Antagonistic effect of strain GX0002980 against plant pathogenic fungi: (**A**) *Bipolaris sorokiniana*, (**B**) *Fusarium pseudograminearum*, (**C**) *Fusarium oxysporum*, (**D**) *Plectosphaerella cucumerina*, (**E**) *Colletotrichum siamense*, (**F**) *Colletotrichum asianum*, (**G**) *Rhizoctonia solani*, (**H**) *Cryphonectria parasitica*, (**I**) *Botryosphaeria dothidea*, (**J**) *Alternaria alternata*, (**K**) *Colletotrichum fructicola*, and (**L**) *Fusarium chlamydosporum*.

The plate confrontation method was used to detect the inhibitory effect of strain GX0002980 on 12 common fungal pathogens of plants. The results found that this strain had the highest inhibition rate against *A. alternata* at 87.29%, and the inhibition rates against the other pathogens ranged from 57.52% to 79.40%, demonstrating broad-spectrum antimicrobial capabilities (Fig. 6; Table 2).

**TABLE 2:** Inhibitory effects of strain GX0002980 on 12 plant pathogens^*a*^

Pathogenic fungi	Inhibition rate (%)	Pathogenic fungi	Inhibition rate (%)
*Bipolaris sorokiniana*	79.39 ± 0.10^b^	*Rhizoctonia solani*	74.55±0.52^c^
*Fusarium pseudograminearum*	65.57 ± 0.12^e^	*Colletotrichum fructicola*	65.28 ± 0.08^e^
*Fusarium oxysporum*	57.53±0.06^g^	*Colletotrichum asianum*	68.94 ± 0.26^d^
*Fusarium chlamydosporum*	61.66±0.16^f^	*Colletotrichum siamense*	67.89 ± 0.20^d^
*Alternaria alternata*	87.31±0.37^a^	*Plectosphaerella cucumerina*	58.26±0.10^g^
*Cryphonectria parasitica*	67.68 ± 0.52^d^	*Botryosphaeria dothidea*	74.80±0.57^c^

^*a*^ Values represent the mean ± SD; different lowercase letters indicate significant differences in the inhibition rates of strain GX0002980 against the fungal pathogens (*P* < 0.05).

### Detection of antimicrobial extracellular enzymes of strain GX0002980

**Fig 7:**
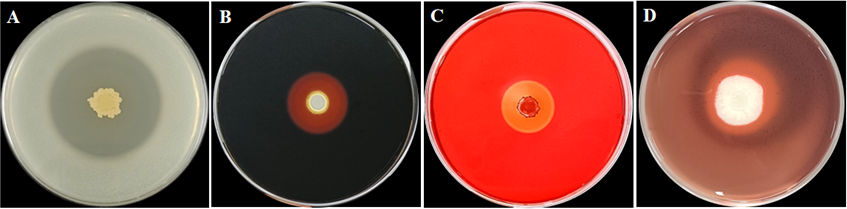
Detection of antimicrobial extracellular enzyme activity of strain GX0002980: (**A**) protease, (**B**) amylase, (**C**) cellulase, and (**D**) pectinase.

Extracellular hydrolytic enzymes are particularly important for the inhibition of pathogens. Therefore, this experiment tested the activity of various extracellular enzymes. The results showed that strain GX0002980 produced large clear zones on the detection media for protease, amylase, cellulase, and pectinase, indicating that strain GX0002980 has the ability to produce proteases, amylases, cellulases, and pectinases (Fig. 7).

### Qualitative detection of the growth-promoting function of strain GX0002980

**Fig 8:**
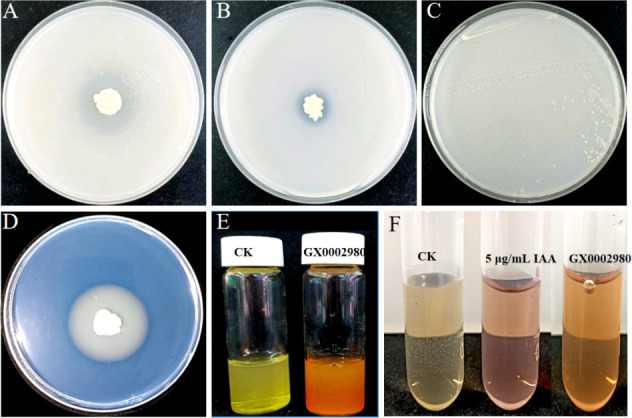
Detection of growth-promoting characteristics of strain GX0002980: (**A**) solubilization of organic phosphorus, (**B**) solubilization of inorganic phosphorus, (**C**) nitrogen fixation, (**D**) siderophore detection, (**E**) production of NH_3_, and (**F**) indole-3-acetic acid (IAA) detection.

Strain GX0002980 produces clear zones on calcium phytate medium (Fig. 8A) and Pikovskaya medium (Fig. 8B), orange-yellow halos on the chrome azurol S assay medium (Fig. 8D), and is capable of growth on Ashby’s nitrogen fixation medium (Fig. 8C). After the addition of Nessler’s reagent, the peptone water medium turns reddish brown with precipitation (Fig. 8E), indicating that GX0002980 has the abilities to solubilize organic phosphorus, solubilize inorganic phosphorus, produce siderophores, fix nitrogen, and produce NH_3_. Additionally, the Salkowski colorimetric assay results show that strain GX0002980 has the ability to secrete IAA, with an IAA content of 7.25 mg/L (Fig. 8F).

### Genome characterization and functional annotation

**Fig 9:**
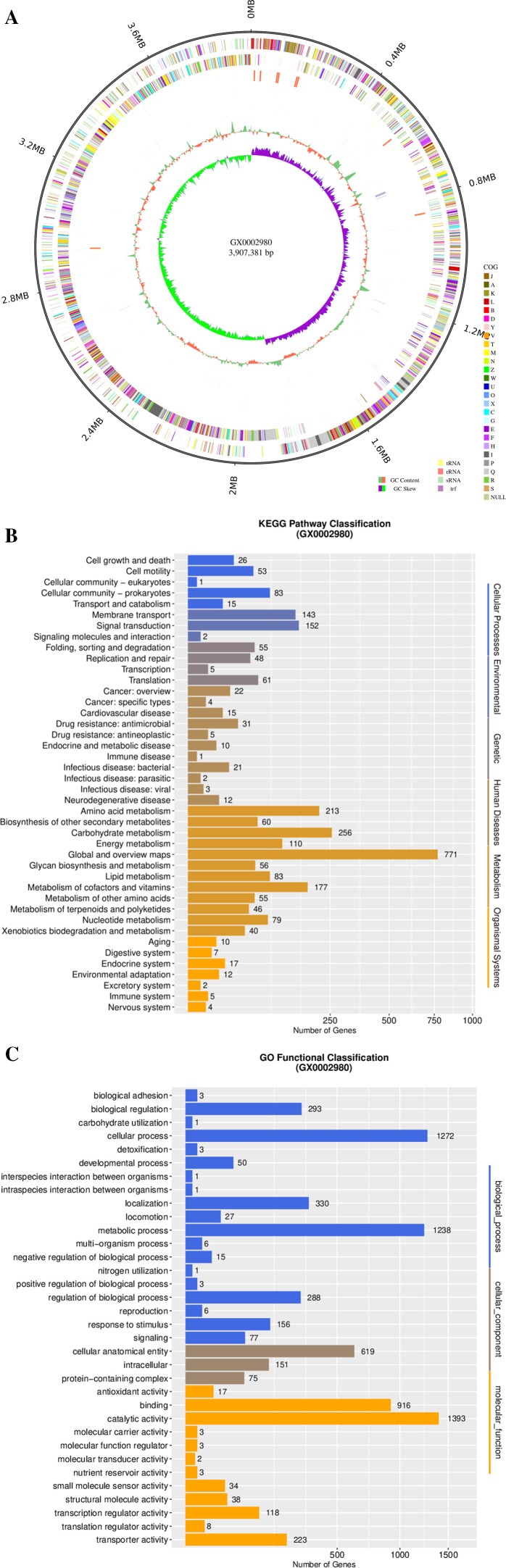
Whole-genome analysis of strain GX0002980: (**A**) circular genome map of strain GX0002980, (**B**) Kyoto Encyclopedia of Genes and Genomes (KEGG) annotation of GX0002980, and (**C**) Gene Ontology (GO) annotation of GX0002980. GC, guanine and cytosine.

The genome of *B. velezensis* GX0002980 is a circular chromosome with a size of 3,907,381 bp. The total length is 3,485,334 bp, with an average gene length of 885.28 bp. The length of the coding genes accounts for 89.20% of the whole genome. The base guanine and cytosine content is 47.44%, with a predicted total of 3,937 genes, out of which 333 are unknown genes. This chromosome contains 87 tRNAs and 27 rRNAs, with 2,962 (75.23%), 2,328 (59.13%), and 2,511 (63.77%) genes respectively matching in the COG, GO, and KEGG databases (Fig. 9A; Table 3).

**TABLE 3:** Genetic features of *B. velezensis* GX0002980

Features	Value
Genome size (bp)	3,907,381
Gene total length (bp)	3,485,334
GC content (%)	47.44
Genes of KEGG	2,511
Genes of COG	2,962
Genes of GO	2,328
tRNA number	87
rRNA number	27
sRNA number	33
ncRNA number	147
Repeat number	307
Gene number	3,937
Contig number	1

To further clarify the taxonomic status of GX0002980 (Table 4), ANI and dDDH analyses were performed. The results showed that GX0002980 had ANI values exceeding 95% and dDDH values above 70% when compared to *B. velezensis* strains 160, FZB42, and LOH117. In contrast, strains from other *Bacillus* spp. exhibited ANI values below 95% and dDDH values below 70%. According to the established species delineation thresholds (ANI ≥95% and dDDH 70%) (27), GX0002980 can be classified as *B. velezensis*, which is consistent with results presented in the section morphological identification and housekeeping gene analysis. Therefore, GX0002980 is confirmed as *B. velezensis*.

**TABLE 4:** The results of ANI and dDDH

Number	Species	ANI (%)	dDDH (%)
GCF 029282585.1	*Bacillus velezensis* 160	98.96	94.60
GCF 000015785.2	*Bacillus velezensis* FZB42	97.61	94.80
GCF 022313395.1	*Bacillus velezensis* LOH112	97.37	87.40
GCF 000196735.1	*Bacillus amyloliquefaciens* DSM 7	93.76	55.50
GCF 012225885.1	*Bacillus tequilensis* EA-CB0015	76.93	21.20
GCF 031317525.1	*Bacillus tequilensis* ATCC^*a*^ BAA-819	76.60	20.90
GCF 002055965.1	*Bacillus subtilis* NCIB 3610	76.38	28.10
GCF 000009045.1	*Bacillus subtilis* subsp. *subtilis* 168	76.36	21.00
GCF 000007825.1	*Bacillus cereus* ATCC 14579	66.93	34.40
GCF 002220285.1	*Bacillus cereus* FORC-047	66.72	36.90
GCF 025947955.1	*Bacillus thuringiensis* Bt Gxmzu777-1	65.94	26.90
GCF 000161615.1	*Bacillus thuringiensis* ATCC 10792	65.90	30.60

^*a*^ ATCC, American Type Culture Collection.

The whole-genome sequencing data of GX0002980 (accession number CP148060) has been uploaded to the NCBI.

### KEGG functional classification annotation

A total of 2,511 genes of *B. velezensis* GX0002980 were annotated to 237 KEGG pathways, among which the most enriched were global and overview maps pathways with 771 genes, carbohydrate metabolism pathways with 256 genes, and amino acid metabolism pathways with 213 genes. A focused analysis on the antibiotic synthesis pathways related to biocontrol in the KEGG pathways of *B. velezensis* GX0002980 showed that *B. velezensis* GX0002980 contains 12 metabolic pathways that can synthesize 13 types of metabolites related to antibiotic synthesis, such as neomycin, kanamycin, gentamicin, novobiocin, carbapenem, streptomycin, ansamycins, penicillin, and cephalosporin (Fig. 9B; Table 5).

**TABLE 5:** Antibiotic pathways related to the enriched pathways in the genome of *B. velezensis* GX0002980

Pathway enrichment genome	Number of enriched genes	Pathway ID
Neomycin, kanamycin, and gentamicin biosynthesis	1	ko00524
Novobiocin biosynthesis	4	ko00401
Streptomycin biosynthesis	9	ko00521
Biosynthesis of ansamycins	1	ko01051
Penicillin and cephalosporin biosynthesis	3	ko00311
Vancomycin resistance	7	ko01502
Biosynthesis of vancomycin group antibiotics	1	ko01055
Biosynthesis of various antibiotics	9	ko00998
Acarbose and validamycin biosynthesis	3	ko00525
Cationic antimicrobial peptide resistance	13	ko01503
Carbapenem biosynthesis	3	ko00332
Prodigiosin biosyntheses	13	ko00333

### GO functional category annotation

The *B. velezensis* GX0002980 genome has a total of 2,328 genes annotated in GO annotation. Among these, the annotations are more numerous in the biological process category, with cellular processes accounting for 1,272 genes and metabolic processes for 1,238 genes; in the cellular component category, cellular anatomical entity includes 619 genes; in the molecular function category, there are 1,393 genes with catalytic activity and 916 genes associated with binding, among others (Fig. 9C).

### Predicted genes for secondary metabolites

The secondary metabolite gene clusters of *B. velezensis* GX0002980 were predicted online using antiSMASH. The results show that *B. velezensis* GX0002980 has 14 secondary metabolite synthesis gene clusters, of which 10 are known gene clusters. These include two belonging to NRPS, three to transAT-PKS, one to NRP-metallophore, one to RRE-containing, one to PKS-like, and one to thiopeptide, involved in the synthesis of surfactin, kijanimicin, plantazolicin, butirosin A/B, macrolactin H, bacillaene, fengycin, difficidin, bacillibactin, and bacillysin. Kijanimicin and plantazolicin show only 4% and 7% similarity, respectively, to known gene sequences, indicating differences from known secondary metabolites. Additionally, two terpene clusters, one lanthipeptide, and one T3PKS synthesis gene cluster did not match any similar gene clusters, suggesting that *B. velezensis* GX0002980 may possess novel gene clusters related to antagonistic substances, demonstrating potential for research and application (Table 6).

**TABLE 6:** Prediction of secondary metabolite synthesis gene clusters in *B. velezensis* GX0002980

Cluster ID	Location (bp)	Cluster type	Similar cluster	Similarity (%)	Suppress object
Cluster 1	305,066–369,875	NRPS	Surfactin	78	Bacteria and fungi
Cluster 2	573,611–602,445	Thiopeptide, LAP	Kijanimicin	4	
Cluster 3	685,849–709,026	RRE-containing, LAP	Plantazolicin	91	
Cluster 4	920,697–961,941	PKS-like	Butirosin A/B	7	Bacteria
Cluster 5	1,046,816–1,064,147	Terpene	–^*a*^	–	–
Cluster 6	1,184,319–1,213,208	Lanthipeptide class II	–	–	–
Cluster 7	1,376,956–1,464,754	TransAT-PKS	Macrolactin H	100	Bacteria
Cluster 8	1,684,264–1,784,978	TransAT-PKS, T3PKS, and NRPS	Bacillaene	100	Bacteria
Cluster 9	1,851,015–1,987,114	NRPS, transAT-PKS, and beta-lactone	Fengycin	100	Fungi
Cluster 10	2,010,675–2,032,558	Terpene	–	–	–
Cluster 11	2,083,068–2,124,168	T3PKS	–	–	–
Cluster 12	2,252,080–2,345,854	TransAT-PKS	Difficidin	100	Bacteria
Cluster 13	3,004,402–3,056,194	NRP-metallophore, NRPS, RiPP-like	Bacillibactin	100	Bacteria and fungi
Cluster 14	3,576,167–3,617,585	Other	Bacillysin	100	Bacteria and fungi

^*a*^ -, no matching items.

**Fig 10:**
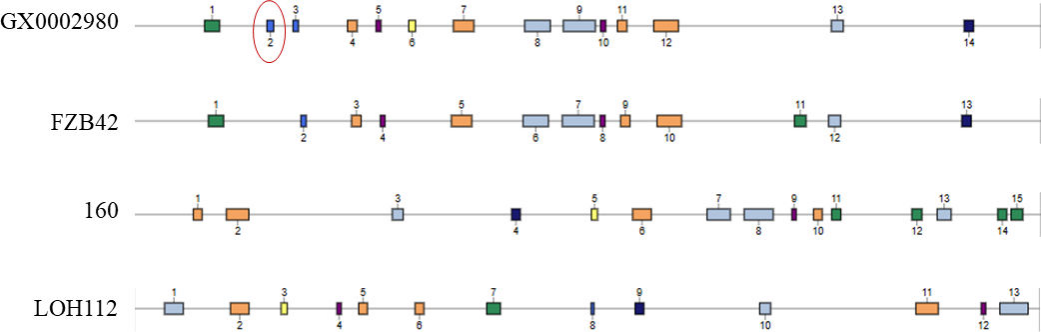
Comparative analysis of secondary metabolite gene clusters.

**Fig 11:**
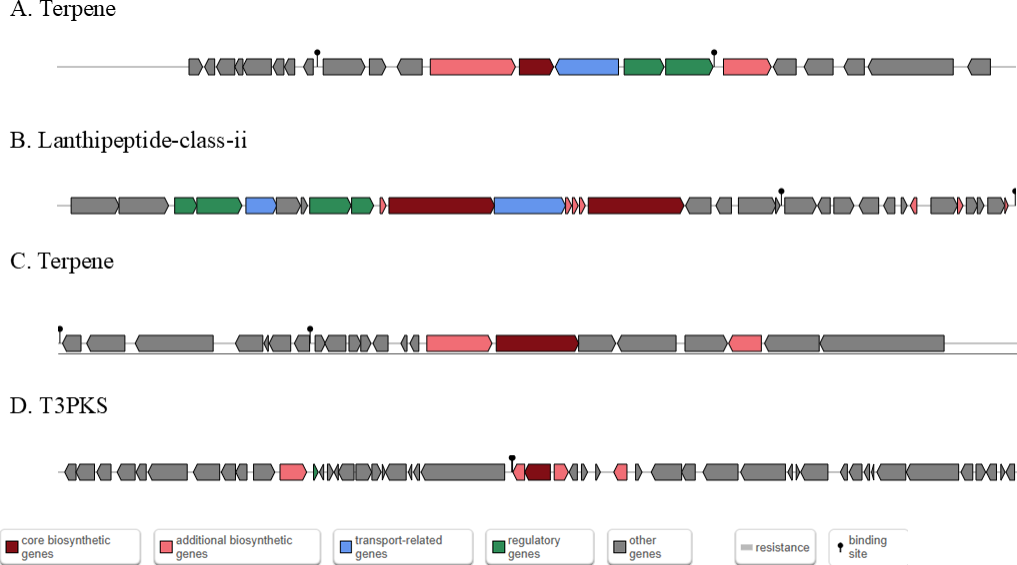
No gene clusters matching secondary metabolites in the database.

A comparative analysis of secondary metabolite gene clusters (Fig. 10) between the GX0002980 strain and previously reported biocontrol strains *Bacillus velezensis* FZB42 (28), 160 (29), and LOH112 (30) revealed that GX0002980 possesses a greater number of secondary metabolite gene clusters than FZB42 and LOH112. This suggests that GX0002980 may produce a wider variety of metabolites and potentially exhibit stronger antibacterial activity. In comparison to strain 160, GX0002980 displays lower similarity in secondary metabolite gene cluster composition, indicating that the two strains may exert antibacterial effects through the production of distinct metabolites. Further comparative analysis showed that the secondary metabolites surfactin, butirosin A/B, macrolacton, bacillaene, fengycin, bacillibactin, and bacilliysin are conserved across FZB42, LOH112, and 160. However, GX0002980 harbors a unique Cluster 2, which encodes a thiopeptide capable of producing kijanimicin. Kijanimicin is a distinctively large acidic enol antibiotic that exhibits *in vitro* activity against certain gram-positive and anaerobic microorganisms (31), with only 4% similarity to known kijanimicin-type compounds in the database. In addition, GX0002980 contains four secondary metabolite gene clusters (Clusters 5, 6, 10, and 11) (Fig. 11) that do not match any known compounds in the database. This suggests that GX0002980 may have the potential to produce novel secondary metabolites, making it a valuable candidate for further research.

### Antimicrobial activity determination and component analysis of strain GX0002980

**Fig 12:**
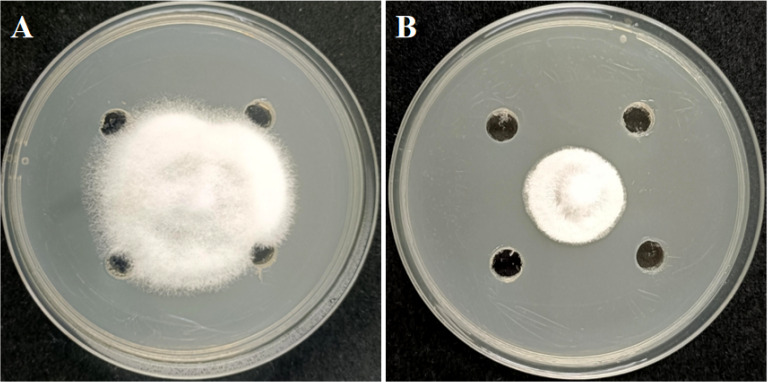
Antimicrobial activity of the ethyl acetate extract from the sterile fermentation broth of *B. velezensis* GX0002980 against *C. gloeosporioides*: (**A**) methanol control and (**B**) ethyl acetate extract.

**Fig 13:**
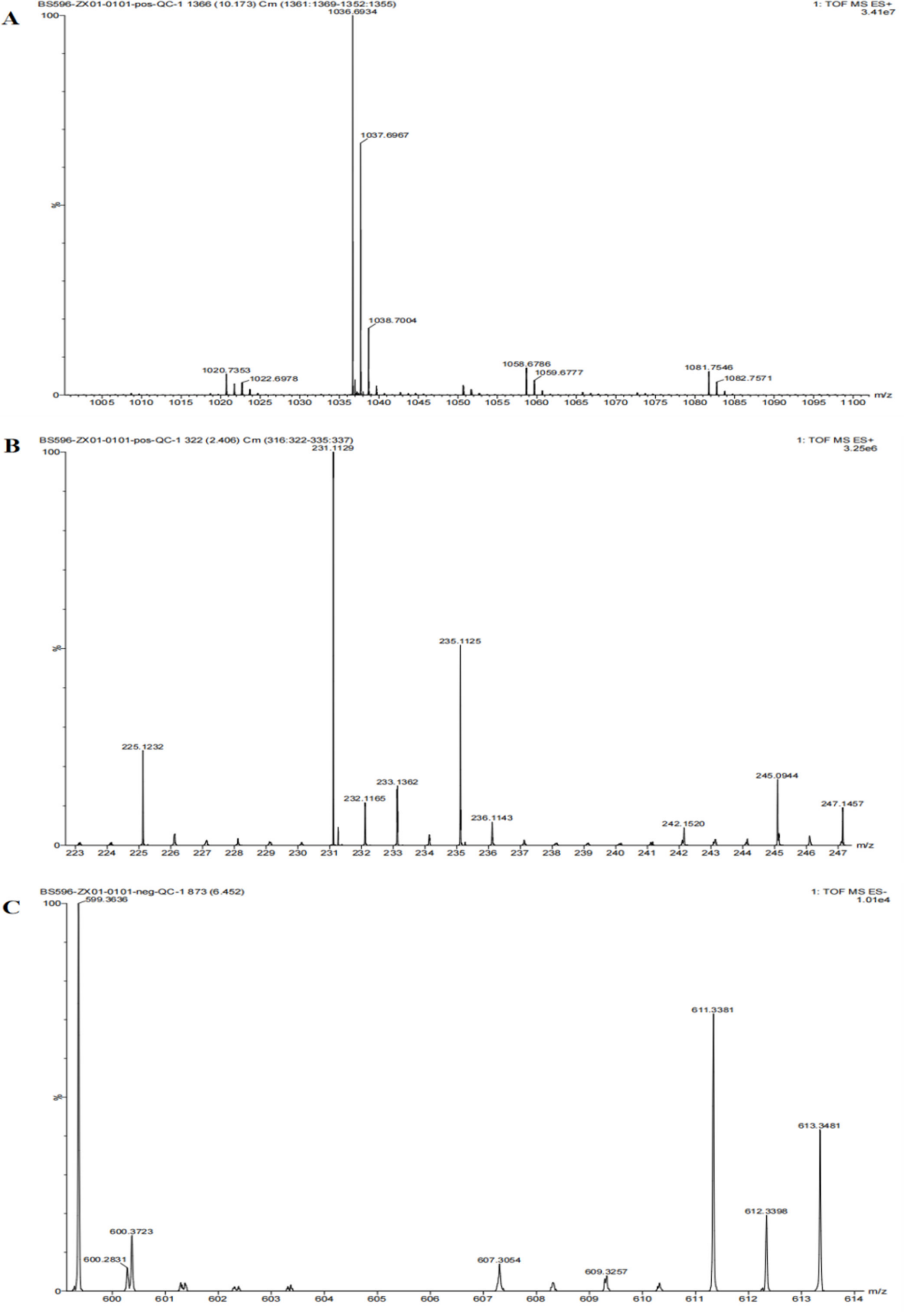
LC-MS analysis of the antimicrobial active substances of *B. velezensis* GX0002980: (**A**) mass spectrum with *m*/*z* values of 1,022.6978 and 1,036.6934; (**B**) mass spectrum with an *m*/*z* value of 235.1125; (**C**) mass spectrum with an *m*/*z* value of 600.2831.

The crude extract of *B. velezensis* GX0002980 with antimicrobial activity was obtained through ethyl acetate extraction combined with rotary evaporation. After being dissolved in a small amount of methanol, its antimicrobial activity was tested and found to be effective against *C. gloesporioides* (Fig. 12). The antimicrobial activity of *B. velezensis* GX0002980 was analyzed by LC-MS, and the antibacterial substance composition was determined. Ions with mass-to-charge ratio (*m*/*z*) values of 1,022.6978 and 1,036.6934 [M + H]^+^ were detected in the crude extract, which are close to the values for C14 surfactin [M + H]^+^ and C15 surfactin [M + H]^+^, respectively. The compounds with *m*/*z* values of 235.1125 and 600.2831 were identified as bacilysin and butirosin A, respectively (Fig. 13).

### Effect of strain GX0002980 on the prevention and treatment of anthracnose in mango and its storage after harvest

**Fig 14:**
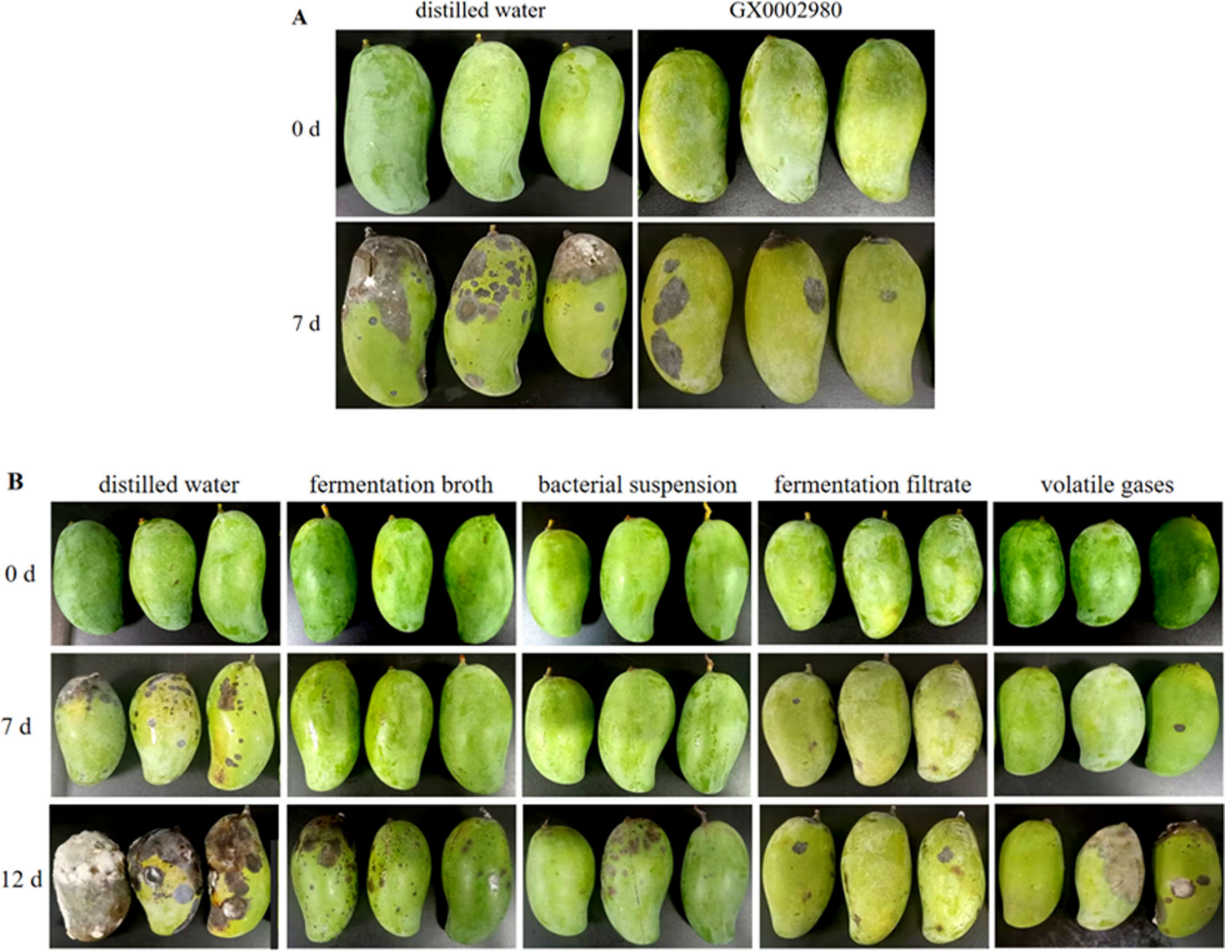
The efficacy of strain GX0002980 against post-harvest anthracnose in mangoes and its effect on post-harvest preservation under natural conditions: (**A**) the control efficiency of strain GX0002980 against post-harvest anthracnose in mangoes and (**B**) the effect of strain GX0002980 on post-harvest preservation of mangoes under natural conditions.

After storing the mango fruits at 25°C for 7 days, strain GX0002980 was able to maintain their appearance quality well. The control group of mangoes, sprayed with the pathogen of mango anthracnose, showed a large area of disease occurrence, while the mangoes treated with the GX0002980 bacterial suspension showed only slight lesions, with a control efficiency against mango anthracnose of 52% (Fig. 14A). At the same time, under natural infection conditions, the fermentation broth, bacterial suspension, fermentation filtrate, and volatile gases of strain GX0002980 could also effectively suppress the occurrence of post-harvest diseases in mangoes, better maintain the quality of mangoes, and extend the post-harvest storage time (Fig. 14B).

## DISCUSSION

*Bacillus* is one of the most common and easily cultured plant growth-promoting rhizobacteria, known for its strong stress resistance and ability to induce plant disease resistance (32, 33). It can produce a variety of antimicrobial active substances, including lipopeptide compounds, polyketone compounds, volatile compounds, and antimicrobial peptides, which have great potential in enhancing agricultural productivity and controlling microbial diseases.

Current research has shown that *B. velezensis* has the ability to inhibit pathogenic microorganisms and promote plant growth (34). In this study, *B. velezensis* GX0002980 exhibited strong inhibitory effects on the mycelial growth of *C. gloesporioides*, with scanning electron microscopy observations revealing shrinkage, distortion, and entanglement of the *C. gloesporioides* mycelia. These phenomena are similar to the mycelial inhibition of the pathogen *C. gloesporioides* by the antagonist *B. velezensis* CE100 reported by Choub et al. (35), which may be due to the antimicrobial proteins produced by GX0002980, such as protease, cellulase, amylase, and pectinase, which damage the cell walls of the fungal pathogen *C. gloesporioides* to achieve an antibacterial effect. As reported by Dhouib et al. (36), *B. velezensis* C2 exerts its antibacterial effect by secreting proteases, chitinases, and β-glucanases to degrade the fungal cell walls. Additionally, the GX0002980 strain also has growth-promoting properties such as nitrogen fixation, NH_3_ production, phosphate solubilization, IAA production, and siderophore production, which are significant for the in-depth exploration of microbial resources for post-harvest disease control in mangoes.

This study isolated a strain from plant rhizosphere soil, *B. velezensis* GX0002980, which exhibits strong antagonistic activity against *C. gloesporioides*. To further investigate the antagonistic and biocontrol effects of *B. velezensis* GX0002980, whole-genome sequencing analysis was performed. AntiSMASH was used to mine potential active metabolic product synthesis gene clusters, with the prediction results showing that the bacteria contain multiple antimicrobial active secondary metabolite synthesis gene clusters. Clusters with higher similarity include lipopeptide compounds surfactin and fengycin; polyketide compounds difficidin, macrolactin H, and bacillaene; as well as secondary metabolites like bacillibactin, bacilysin, and plantazolicin, all of which have significant antibacterial activity against bacteria or fungi. *B. velezensis* mainly exerts its antibacterial effects through the production of lipopeptide and polyketide antibiotics (37). The antibacterial mechanism of lipopeptide substances primarily targets the cell wall and membrane, and lipopeptide compounds surfactin and fengycin possess broad-spectrum antibacterial activity and exhibit excellent performance in inhibiting fungal growth (38). Surfactin has attracted widespread attention due to its significant surfactant capability and its combined antifungal, antimycoplasma, and antiviral effects (39, 40). Polyketone antibiotics have been proven to have a strong inhibitory effect on a variety of plant pathogenic bacteria and can enhance the resistance of plants (41). Bacilysin is a dipeptide antibiotic compound with broad inhibitory activity against fungi and bacteria. Its synthesis pathway within microbes is quite complex, making it a hot compound in the field of natural products in recent years (42). Bacillibactin is a siderophore with a strong binding affinity for Fe^3+^, limiting the supply of iron required for normal metabolism to inhibit the growth of pathogens (43). Kijanimicin and butirosin A/B, with only 4% and 7% similarity, respectively, have antagonistic effects against gram-positive and gram-negative bacteria (44). Therefore, it can be inferred that the antagonism of strain GX0002980 against pathogenic fungi may mainly come from these substances with known excellent antibacterial activity. Meanwhile, some other unidentified metabolites may also have potential antibacterial activity or other biological functions.

It was found that the metabolites of *B. velezensis* contained a variety of antibacterial substances. Zhou et al. (45) have found that *B. velezensis* BR-01 contained surfactin, iturin, and fengycin and other active substances by LC-MS detection and analysis technology, which had a good control effect on a variety of rice diseases. In this study, surfactin, bacilysin, and butirosin A were the main antibacterial components of *B. velezensis* GX0002980, which were consistent with the predicted gene clusters for secondary metabolites in the GX0002980 genome. For lipopeptide antibiotics, only surfactin was detected in the crude extract of fermentation filtrate of GX0002980, while fegycin was not detected. According to the literature, fegycin has broad-spectrum antagonistic activity against filamentous fungi (46, 47). Meanwhile, the antiSMASH detected the presence of the fengycin gene cluster. This may be due to the fact that fengycin detection peaks only appear within the *m*/*z* range of 1,450–1,550 and due to experimental instrument limitations that can only detect substances within the *m*/*z* range of 50–1200, resulting in fengycin not being detected.

*B. velezensis* is widely used in the biological control and preservation of post-harvest fruit diseases (48). The *B. velezensis* GX0002980 strain selected in this study has shown significant effects in the prevention and treatment of mango anthracnose. Its fermentation broth, fermentation filtrate, bacterial suspension, and volatile gases all have preventive and treatment effects on post-harvest anthracnose of mangoes, effectively inhibiting the occurrence of post-harvest anthracnose in mangoes, better maintaining mango quality, and extending the post-harvest storage time of mangoes.

Evaluating the field efficacy of *B. velezensis* and its natural products has been a key research strategy in recent years (49). However, field experiments are constrained by the growth cycle of mango trees, fluctuations in the natural environmental conditions, and the actual application forms of biocontrol agents. The use of microbial natural products for field control is considered a more environmentally friendly and safer approach. Therefore, in future research, we aim to further identify the antibacterial active compounds responsible for the inhibitory effects of GX0002980 and utilize engineered bacteria to induce or enhance their production. These bioactive compounds could then be applied as microbial natural products for field control. The impact of GX0002980 on mango fruit can be evaluated using disease indices, including the incidence rate per mango tree, the probability of disease spot formation, and physiological changes in harvested mango fruit.

However, previous studies have indicated that excessive use of *Bacillus*-based biocontrol agents may have unintended consequences, such as negative effects on non-target microorganisms, induction of plant stress responses, or disruption of the plant-associated microbial community balance (50, 51). Therefore, in practical applications, it is crucial to assess the ecological impact and physiological balance of plants under field conditions, determine the optimal application concentration, and ensure the safety of biocontrol formulations.

Volatile organic compounds produced by microbes have great potential in the control of post-harvest fruit diseases due to their high antibacterial efficiency, biodegradability, and absence of residue on fruit surfaces (52–54). This study preliminarily explored and found that the volatile substances produced by the *B. velezensis* GX0002980 strain have antagonistic effects against *C. gloesporioides* and preventive effects against post-harvest anthracnose in mangoes. However, this study has not yet conducted in-depth research on the antimicrobial mechanism of which volatile substances produced by the GX0002980 strain play a major role in biocontrol. This will also be a focus of subsequent research.

### Conclusions

In this study, *B. velezensis* GX0002980 was isolated from the rhizosphere soil of plants, which showed a strong inhibitory effect on *C. gloesporioides*. The sterile fermentation filtrate and volatiles produced by this bacterium also have an antagonistic effect on *C. gloesporioides* and maintain the post-harvest quality of mango fruits. Strain GX0002980 produced a variety of hydrolytic enzymes, which caused the mycelia of pathogenic bacteria to wrinkle, distort, and tangle, and inhibited the mycelia growth of *C. gloesporioides*. Whole-genome sequencing results showed that GX0002980 had a variety of inhibitory secondary metabolite synthesis gene clusters. The antibacterial substances of crude extract of GX0002980 were analyzed by LC-MS technology. The antibacterial substances were C14 surfactin [M + H]^+^, C15 surfactin [M + H]^+^, bacilysin, and butirosin A. It is basically consistent with antiSMASH in predicting the synthesis gene cluster of bacteriostatic secondary metabolites. These gene clusters and secondary metabolites are critical in the broad-spectrum inhibition of pathogenic bacteria. Therefore, strain GX0002980 has great application potential in the biological control of mango anthracnose.

## Data Availability

Data will be made available on request.
